# Chemogenomics knowledgebased polypharmacology analyses of drug abuse related G-protein coupled receptors and their ligands

**DOI:** 10.3389/fphar.2014.00003

**Published:** 2014-02-06

**Authors:** Xiang-Qun Xie, Lirong Wang, Haibin Liu, Qin Ouyang, Cheng Fang, Weiwei Su

**Affiliations:** ^1^Department of Pharmaceutical Sciences, Computational Chemical Genomics Screening Center, School of Pharmacy, University of PittsburghPittsburgh, PA, USA; ^2^Center for Chemical Methodologies and Library Development (UPCMLD) and Department of Chemistry, University of PittsburghPittsburgh, PA, USA; ^3^Drug Discovery Institute, University of PittsburghPittsburgh, PA, USA; ^4^Departments of Computational and Systems Biology, University of PittsburghPittsburgh, PA, USA; ^5^Guangzhou Quality R&D Center of Traditional Chinese Medicine, School of Life Sciences, Sun Yat-Sen UniversityGuangzhou, China

**Keywords:** drug abuse, GPCRs, polypharmacology, polydrug addiction, chemogenomics, cloud computation, target prediction, systems pharmacology

## Abstract

Drug abuse (DA) and addiction is a complex illness, broadly viewed as a neurobiological impairment with genetic and environmental factors that influence its development and manifestation. Abused substances can disrupt the activity of neurons by interacting with many proteins, particularly G-protein coupled receptors (GPCRs). A few medicines that target the central nervous system (CNS) can also modulate DA related proteins, such as GPCRs, which can act in conjunction with the controlled psychoactive substance(s) and increase side effects. To fully explore the molecular interaction networks that underlie DA and to effectively modulate the GPCRs in these networks with small molecules for DA treatment, we built a drug-abuse domain specific chemogenomics knowledgebase **(DA-KB)** to centralize the reported chemogenomics research information related to DA and CNS disorders in an effort to benefit researchers across a broad range of disciplines. We then focus on the analysis of GPCRs as many of them are closely related with DA. Their distribution in human tissues was also analyzed for the study of side effects caused by abused drugs. We further implement our computational algorithms/tools to explore DA targets, DA mechanisms and pathways involved in polydrug addiction and to explore polypharmacological effects of the GPCR ligands. Finally, the polypharmacology effects of GPCRs-targeted medicines for DA treatment were investigated and such effects can be exploited for the development of drugs with polypharmacophore for DA intervention. The chemogenomics database and the analysis tools will help us better understand the mechanism of drugs abuse and facilitate to design new medications for system pharmacotherapy of DA.

## Introduction

Drug abuse (DA) and addiction is a complex brain disease characterized by compulsive drug craving, seeking, and use that persist in spite of severe adverse consequences. The basic mechanisms of DA have been explored for decades. It is well accepted that abused substances can disrupt activity of normal nerve cells by interacting with receptors in the brain, like G-protein coupled receptors (GPCRs), and then activating the downstream signaling pathways, which comprise a host of kinases and transcription factors. Specifically, there is a broad range of GPCRs related to the DA and central nervous system (CNS) side effects, including receptors of opioid, cannabinoid, and Serotonin. Further, it is estimated that genetic factors play a significant role, approximately 40–60% of the total risk (Kendler et al., 2008). At least 396 genes related to DA were summarized by researchers in 2008 (Li et al., 2008). Additional genes were found to be associated with DA by NIDA genetics experts and other individual scientists (Saccone et al., 2008, 2009). Indeed, studies on DA, CNS diseases, and relevant research have been steadily proliferating. Concomitantly, the quantity and quality of molecular therapies for DA and CNS disorders are advancing, thanks in part to rapid technology development in biochemistry, biophysics, and pharmacology, as well as the breakthrough discoveries of opioid-ligand co-crystallographic structures (Granier et al., 2012; Manglik et al., 2012; Thompson et al., 2012; Wu et al., 2012). Unfortunately, the venues or reports that publicize DA and neuromedicine research are scattered in journals, periodicals, or in databases, such as SciFinder, PubChem, ChEMBL (Bender, 2010; Gaulton et al., 2012), and DrugBank (Wishart et al., 2006, 2008; Knox et al., 2011). It is inconvenient to find, associate and validate reported DA and neuro-active molecules and reuse the reported results for further studies. It is thus essential to centralize reported chemogenomics research data related to DA chemicals and CNS medications in order to benefit researchers across broad disciplines.

Today, a few medications have been developed and used in the clinic for DA treatment, and most of them are targeting at GPCRs or transmembrane proteins in brain CNS system. For example, methadone, buprenorphine, and naltrexone are available for individuals addicted to opioids, whereas nicotine preparations and medications (varenicline and bupropion) are available for treatment of tobacco addiction. Some unintended consequences of these treatments, however, are causes for concern. For example, long-term methadone treatment can cause negative changes in the brain, according to recent studies (Andersen et al., 2012; Sankararaman et al., 2012). Methadone use accounted for more than 30 percent of overdose deaths from prescription painkillers in the US, despite accounting for only 2 percent of pain prescriptions as reported by the Center for Disease Control and Prevention (CDC, 2014). As such, there remains a great demand for design and discovery of new medications for the treatment of DA by targeting at GPCRs.

Another major challenge in the development of CNS drugs for DA treatment is the polypharmacology associated with GPCRs and other CNS targeting molecules. **Polypharmacology** is defined as the activity of compounds on multiple targets (Peters, 2012). Methadone, for example, can interact with at least the μ, δ, and κ opioid receptors, an important rhodopsin or class A GPCR family. Likewise, a number of medicines that target the CNS diseases can also modulate the DA-related proteins, which can act in concert with controlled psychoactive substances and increase side effects. The atypical antipsychotic drug clozapine, for example, shows antagonist activity at multiple aminergic GPCR family, such as the 5HT, dopamine, muscarinic, histamine, and adrenergic receptors, some of which are associated with efficacy and others with side effects (Morphy et al., 2004; Morphy, 2012). More examples can be found in the current literature (Kroeze and Roth, 2012). Another important consideration is that DA and addiction often co-occur with other mental illnesses (Regier et al., 1990). When an individual experiences addiction to more than one drug at the same time (so called **polydrug addiction**), he/she carries more risk than one addicted to a single drug, due to an increase in side effects and drug synergy. Data from the DA Warning Network indicate that the majority of patients with prescriptions for benzodiazepines or opioids who are admitted to the emergency room had recently used one or more other substances, most frequently alcohol (SAMHSA, 2009). The ideal treatment should therefore break the addiction to each drug. The design of medicines for DA treatment must address the polypharmacology problem through: (i) lessening unwanted off-target activities that may lead to adverse drug reactions; and (ii) designing polypharmacological drugs with multiple activities across DA-related target classes, such as GPCRs, ion channels, or kinases for improved therapies (Peters, 2012). It is advantageous to utilize a polypharmacological approach in this manner to treat polygenic diseases, such as DA, in which a network of disease-related targets are modulated, as opposed to “switching” a single target on or off. Indeed, we have developed and implemented chemical genomics as a novel, powerful tool to address polydrug addiction networks related to these complicated concerns and challenges.

In this manuscript, we reported an integrated platform of cloud computing and cloud sourcing DA chemogenomics knowledgebase (DA-KB) for CNS system pharmacotherapy and new medicinal drug discovery (www.CBLigand.org/DAKB, or www.CBLigand.org/CloudDA). Domain-specific DA-KB database and polypharmacological target identification methods were constructed using our recently published chemogenomics based TargetHunter program (Wang et al., 2013), and GPU-accelerated machine learning/cheminformatics/bioinformatics algorithms and tools (Ma et al., 2011a,b, 2013; Myint et al., 2012). As reported, chemical genomics (or **chemogenomics**) is an interdisciplinary research field that utilizes chemicals/drugs and associated genomics data produced by *in vitro* and *in silico* techniques to systematically identify, analyze and/or predict chemicals-protein interactions for the purpose of enhancing new medicine design. Computational chemogenomics draws from the chemoinformatics and bioinformatics disciplines to produce useful information systems for researchers in pursuit of chemogenomics data, predictive modeling, as well as techniques in ligand- and structure-based drug design (Jacoby, 2011). It transforms the one-target-one-drug development process to a new multi-target-multi-drug paradigm; as such it is appropriate for the study of polydrug polypharmacology networks of DA chemicals and their analogs related to DA-targeted proteins. We have applied our developed chemogenomics database and machine-learning algorithms/tools above, and aimed at polypharmacology prediction for DA research and treatment through cloud computing and sourcing services. Herein, we reported our DA domain-specific chemogenomics knowledgebase (KB) by data-mining literature and public databases to archive DA chemical molecules/substances and the protein targets associated DA signaling pathways. Specifically, we analyzed DA related GPCRs and the poly-pharmacophore effects of their ligands. This information together with our established computational technologies/tools can enable us to further analyze the DA in molecular and systems pharmacology levels, and help us to understand better the DA mechanism of actions. Ultimately, the constructed computational chemogenomics knowledgebase databases and developed computational technologies/tools will help bridge the knowledge gap between chemical structures and their biological activities related to DA, and assist scientists to conduct polypharmacological target identification and design better medicines for DA intervention for translational systems pharmacotherapy and personalized medicine research.

## Materials and methods

### Drug abuse (DA) domain-specific chemogenomics knowledgebase (*DA-KB*)

We have data-mined DA related genes, proteins and compounds as well as associated signaling pathways from literature and public databases, and then compiled them into the **DA-KB** database. In addition, FDA approved CNS drugs also are included in order to study drug repurposing and the possible side effects or off-target effects, as many of these therapeutic drugs can also potentially interact with the DA-related proteins.

The **DA-KB** contains genes, proteins, chemical compounds, and bioassays related to DA and neuropharmacology. The current version of DA-KB consists of the following records:

- DA Genes/Proteins (current: 594 records): the reported genes related to DA (e.g., GPCRS, transporters, ion channels, and kinases) have been data-mined from literature and public databases, e.g., Knowledgebase for Addiction Related Genes (KARG, karg.cbi.pku.edu.cn/), and NIDA Center for Genetic Studies (nidagenetics.org/Results/cand_genes.xls). These genes were then mapped onto proteins.
- DA Chemicals (current: 0.5 million records): these DA-related chemicals/substances are the known US controlled Illicit drugs and/or have been reported biologically to interact with the proteins above. All of them have been archived from journal articles, patents and public repositories such as PubChem and ChEMBL (Bender, 2010; Gaulton et al., 2012), using our established data-mining protocols and controlled vocabulary/ontology. Among these compounds, 197 of US Illicit drugs are included. We also incorporate the CNS drugs reported in the DrugBank database and the PubChem library for later study of polypharmacology and drug repurposing (Ashburn and Thor, 2004; Oprea et al., 2011).
- DA Pathways (current: 594 records): The corresponding signaling pathways for DA proteins have been mapped through public databases, such as KEGG (Kanehisa and Goto, 2000; Kanehisa et al., 2012) and DrugBank (Wishart et al., 2006, 2008; Knox et al., 2011) using our established data mining analysis tools.
- DA Bioassays (current: 49,482 records): The bioassays that can be used to validate the DA target predictions/modeling have been collected from literature and public resources, such as ChEMBL and PubChem (Xie, 2010). Such information on bioassay validation is certain to enhance collaborations among the broad scientific community.

**Figure 1:**
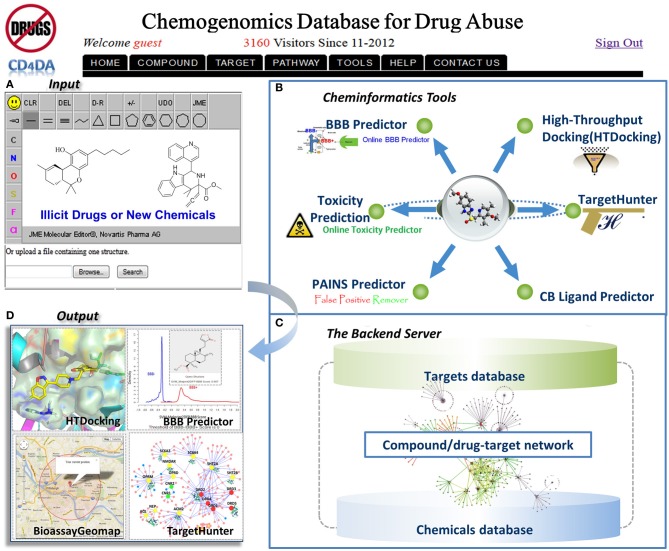
**The overview of our integrated DA domain-specific cloud computing and sourcing platform (CloudDA) with illustration of a friendly user-interfaced query (A) and output (B) and server backend (C) of the constructed computational chemogenomics database as well as the implemented computing tools/programs (D) for DA research**. CloudDA provides the DA chemogenomics knowledgebase, polydrug addiction/polypharmacology prediction tools, and additional online services.

DA-KB is not a new-born database. Actually it was rooted from our established web-interfaced cannabinoid molecular information database (CBID) repository (www.CBligand.org/cbid) (Yang et al., 2012), which was constructed using MySQL/PHP and implemented with our in-house cheminformatics tools/data-mining algorithms. The current DA-KB platform is disseminated via cloud sourcing server (www.CBLigand.org/CloudDA). The current online cheminformatics tools/programs under the GPU-acceleration computing (Ma et al., 2011a) include Target/Off-Target Predictors (TargetHunter and HTDocking) (Wang et al., 2013; HTDocking, 2013) for polydrug addiction/toxicity prediction, artificial neural network (ANN)-QSAR (Myint et al., 2012) and a blood-brain-barrier (BBB) predictor (Wang and Xie, 2010). All these have been integrated to meet the needs of the broad DA research community (Figure 1).

### HTDocking for DA polydrug addiction analysis

We have established a high-throughput docking program (HTDocking, 2013, www.cbligand.org/HTDocking), which is a web-based computing tool that automates docking procedure to search for protein targets and explore ligand/protein interactions. In addition, we also published online high-throughput GPCRs docking program (GPCRDocking, www.CBLigand.org/GPCRHTDocking) to facilitate DA GPCRs target/off-target mapping analysis. In the current version of DA-KB database, 3888 crystal structures of DA-related proteins have been collected from the Protein Data Bank (PDB) (Berman et al., 2000) to build a DA domain-specific subset by searching with sequences from our DA-KB. Water molecules and ligands were removed, and the active site(s) of each protein were defined by the residues around the co-crystalized ligands or manually generated using our published method (Chen et al., 2007). Docking scores are used to assess and rank the potential protein partners of a queried compound. In this study, HTDocking and GPCRDocking programs were used to perform polypharmacology/polydrug addiction analysis for DA related GPCRs, other proteins and their ligands.

### Targethunter for DA for polypharmacology

TargetHunter web server has been developed and used in collaboration with the NIGMS-funded UPCMLD center (Brummond et al., 2012). It has also been employed to predict the off-targets of a class of novel PKD1 inhibitors, which has been further validated by experiments (Tandon et al., 2012). The detailed description of the TargetHunter algorithm has been recently published (Wang et al., 2013). The underlying principle of the TargetHunter program is based on a known medicinal chemistry concept: structurally similar compounds have similar physical properties that may result in similar biological profiles (Martin et al., 2002; Pozzan, 2006; Xie and Chen, 2008). In our reported studies using the public source databases, e.g., ChEMBL and PubChem, the prediction accuracy was 91.1% in a subset of ChEMBL (Wang et al., 2013). TargetHunter is a powerful cloud computing tool with attractive features: (i) ease of use; (ii) query data retrieval function; (iii) user choices of desired fingerprints and databases; and (iv) high accuracy. Importantly, we have innovatively implemented a BioassayGeoMap program to facilitate users, simply via a click “Bioassay Finder,” to easily find the laboratories who have published bioassay(s) for biological validation. Such an integrated tool will assist researchers to conduct target/off-target identification and develop bioactive compounds for DA treatment as illustrated below.

## Results and discussion

### GPCRs and drug abuse

The ***DA-KB*** database provides chemogenomics data information and integrated cheminformatics tools/programs to allows us transform data to knowledge by analyzing the DA in both molecular and system pharmacology levels. For example, it is reported that the nucleus accumbens (NAc) and the ventral tegmental area (VTA) are the primary sites where drugs of abuse act (Nestler, 2001). Commonly abused drugs interact with receptors such as GPCRs (Table 1) on the cell surface and alter the dopamine system by prolonging the action of dopamine in the NAc or by potentiating the activation of neurons in the VTA and NAc. As such, we first analyzed the GPCRs and DA based on the established **DA-KB**. Table 1 lists the 85 GPCRs that are reported to be closely related to DA and CNS system, including well-known cannabinoid receptors (CB1 and CB2), dopamine receptors (D_1−5_), histamine receptors (H_1−4_), opioid receptors (Mu, Delta and Kappa), and glutamate receptors (Metabotropic glutamate receptor _1−8_). Either the proteins can directly interact with abused drugs or their mutations in the genes are reported to associate with DA disorders. For example, the morphine activates opioid receptors in the VTA, NAc, and cerebral cortex, results in feelings of reward and activates the pleasure circuit by causing greater amounts of dopamine to be released within the nucleus accumbens (Nestler and Malenka, 2004). Cannabinoids stimulate CB1 receptor (which, like D2 and opioid receptors, are Gi linked) on glutamatergic and GABAergic nerve terminals in the NAc, and on NAc neurons themselves (Nestler and Malenka, 2004). In addition, the endocannabinoid contents can be altered by chronic exposure to nicotine, ethanol or cocaine (González et al., 2002).

**Table 1:** **List of drug-abuse related GPCRs in the DA-KB database**.

**GPCR name**	**^a^UniProt accession**	**^b^Reported compounds**	**^c^Reported bioactivities**	**^d^Cited references**	**^e^Abused drug/medication**
Adenosine A2a receptor	P29274	9253	311	6466	Adenosine
Adenosine A2b receptor	P29275	4214	151	3003	
Adenosine A3 receptor	P33765	9104	298	5550	
Alpha-1d adrenergic receptor	P25100	3365	113	2072	Lisdexamfetamine^*^
Alpha-2a adrenergic receptor	P08913	3259	174	1831	Benzphetamine^*^
Alpha-2b adrenergic receptor	P18089	2622	86	1375	Methamphetamine^*^
Alpha-2c adrenergic receptor	P18825	2912	102	1503	Methamphetamine^*^
Angiotensin II type 2 (AT-2) receptor	P50052	2255	54	1323	
Beta-1 adrenergic receptor	P08588	4909	234	3025	
Beta-2 adrenergic receptor	P07550	6515	208	4320	
Beta-3 adrenergic receptor	P13945	4925	106	2454	
Bradykinin B2 receptor	P30411	3114	78	1655	
Cannabinoid CB1 receptor	P21554	9380	272	5553	Nabilone^*^
Cannabinoid CB2 receptor	P34972	7760	256	4587	Nabilone^*^
C-C chemokine receptor type 1	P32246	1085	78	727	
C-C chemokine receptor type 2	P41597	4594	100	2500	
C-C chemokine receptor type 4	P51679	2386	46	1300	
C-C chemokine receptor type 5	P51681	4731	133	2931	
Cholecystokinin A receptor	P32238	3612	105	2081	
Cholecystokinin B receptor	P32239	2375	125	1743	
C-X-C chemokine receptor type 3	P49682	1318	34	864	
C-X-C chemokine receptor type 4	P61073	934	58	540	
Cysteinyl leukotriene receptor 1	Q9Y271	2631	65	1505	
Delta opioid receptor	P41143	9268	442	5527	Heroin^*^
Dopamine D1 receptor	P21728	4187	211	2365	(R)-Apomorphine^*^
Dopamine D2 receptor	P14416	10996	590	6945	Ketamine^*^
Dopamine D3 receptor	P35462	6497	356	4289	(R)-Apomorphine^*^
Dopamine D4 receptor	P21917	5207	240	3261	(R)-Apomorphine^*^
Dopamine D5 receptor	P21918	513	77	378	
Endothelin receptor ET-A	P25101	3839	110	2602	
Histamine H1 receptor	P35367	4037	267	2717	Diphenhydramine
Histamine H2 receptor	P25021	2596	154	1585	
Histamine H3 receptor	Q9Y5N1	5265	214	3359	
Histamine H4 receptor	Q9H3N8	2278	118	1357	
Interleukin-8 receptor A	P25024	2036	33	1142	
Interleukin-8 receptor B	P25025	2412	58	1465	
Kappa opioid receptor	P41145	9142	363	5066	
Melanocortin receptor 1	Q01726	1747	82	865	
Melanocortin receptor 2	Q01718	4	1	1	
Melanocortin receptor 3	P41968	3641	89	1864	
Melanocortin receptor 4	P32245	7510	143	3869	
Melanocortin receptor 5	P33032	3238	72	1647	
Mu opioid receptor	P35372	9402	396	5600	Morphine^*^
Muscarinic acetylcholine receptor 1	P11229	6083	282	3646	Cocaine^*^
Muscarinic acetylcholine receptor 2	P08172	5083	274	3236	Cocaine^*^
Muscarinic acetylcholine receptor 3	P20309	5345	250	3410	
Muscarinic acetylcholine receptor 4	P08173	3174	149	1879	
Muscarinic acetylcholine receptor 5	P08912	2900	114	1736	
Neurokinin 1 receptor	P25103	5328	227	3484	
Neurokinin 2 receptor	P21452	3400	131	2162	
Neuropeptide FF receptor 1	Q9GZQ6	229	4	94	
Neuropeptide Y receptor type 1	P25929	3187	89	2066	
Neuropeptide Y receptor type 2	P49146	2585	63	1598	
Neuropeptide Y receptor type 4	P50391	88	23	67	
Neuropeptide Y receptor type 5	Q15761	1453	62	1227	
Nociceptin receptor	P41146	2797	87	1488	
Platelet activating factor receptor	P25105	3787	64	2380	
Serotonin 1a (5-HT1a) receptor	P08908	6461	452	3967	PMA^*^
Serotonin 1b (5-HT1b) receptor	P28222	2099	198	1497	Bufotenin^*^
Serotonin 1d (5-HT1d) receptor	P28221	2359	181	1456	DOET^*^
Serotonin 1e (5-HT1e) receptor	P28566	221	65	218	
Serotonin 1f (5-HT1f) receptor	P30939	199	31	146	
Serotonin 2a (5-HT2a) receptor	P28223	5884	373	4034	Psilocin^*^
Serotonin 2b (5-HT2b) receptor	P41595	3775	196	2227	Mescaline^*^
Serotonin 2c (5-HT2c) receptor	P28335	6102	313	3957	Psilocybin^*^
Serotonin 4 (5-HT4) receptor	Q13639	1192	78	549	
Serotonin 5a (5-HT5a) receptor	P47898	675	106	639	Lysergide^*^
Serotonin 6 (5-HT6) receptor	P50406	5857	232	3462	Lysergide^*^
Serotonin 7 (5-HT7) receptor	P34969	1899	215	1448	
Thyroid stimulating hormone receptor	P16473	29856	2	17114	
Trace amine-associated receptor 1	Q96RJ0	266	6	148	Amphetamine^*^
Vasopressin V1a receptor	P37288	3037	90	1875	
Calcitonin receptor	P30988	1803	6	890	
Glucagon-like peptide 1 receptor	P43220	107911	24	105298	
Vasoactive intestinal polypeptide receptor 1	P32241	1768	14	893	
GABA-B receptor	Q9UBS5	143	7	77	Amobarbital^*^
Metabotropic glutamate receptor 1	Q13255	961	90	754	JNJ16259685
Metabotropic glutamate receptor 2	Q14416	1201	86	747	LY341495
Metabotropic glutamate receptor 3	Q14832	241	37	149	LY341495
Metabotropic glutamate receptor 4	Q14833	1063	60	572	LY341496
Metabotropic glutamate receptor 5	P41594	2637	103	1742	MPEP
Metabotropic glutamate receptor 6	O15303	218	26	181	
Metabotropic glutamate receptor 7	Q14831	125	30	83	
Metabotropic glutamate receptor 8	O00222	142	29	114	

* US Schedule controlled substances.

a UniProt Accession is from UniProt database(http://www.uniprot.org/)

b Number of Reported Compounds.

c Number of Reported Bioactivities.

d ^d^Number of Cited References.

e Represented Abused Drug/Medication.

PMA, 4-methoxyamphetamine or para-Methoxyamphetamine; DOET, 2,5-Dimethoxy-4-ethylamphetamine; MPEP, 2-Methyl-6-(phenylethynyl)pyridine.

The over-the-counter anti-emetic diphenhydramine (DPH) has been reported to be abused for non-medicinal purposes. DPH is an H1 histamine receptor antagonist, and its abuse liability was confirmed as it interacts either directly or indirectly with neurotransmitter systems, including those using acetylcholine, serotonin, norepinephrine, dopamine, opioids or adenosine (Halpert et al., 2002). These drugs will eventually activate CREB and ΔFosB in the NAc, and will cause the cellular adaptations. On the other hand, antagonism of metabotropic glutamate 1 receptors attenuates behavioral effects of cocaine and methamphetamine in squirrel monkeys (Achat-Mendes et al., 2012) and activation of CB2 receptors by CB2 agonists within dopamine terminals can inhibit dopamine release (Xi et al., 2011; Morales and Bonci, 2012), which implies the potential of designing molecules targeting these GPCRs for DA treatment (Achat-Mendes et al., 2012; Yang et al., 2012).

**Figure 2:**
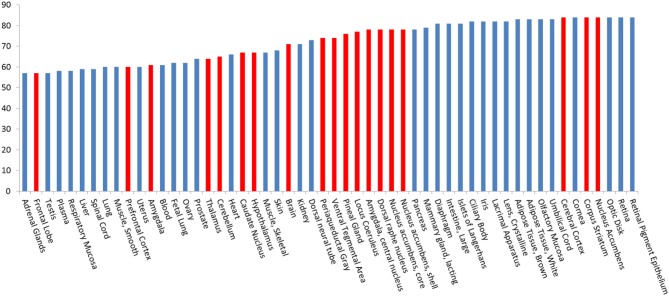
**Tissue distribution of 85 drug-abuse related GPCRs**. Red lines indicate that these tissues are located in the central nervous system (CNS). For example, 84 of these 85 GPCRs are expressed in the nucleus accumbens, where most drugs of abuse act.

The distribution information of 85 drug-abuse related GPCRs in the human body were also collected from the MetaCore database. Figure 2 shows the statistical abundance of tissue distribution of these receptors. Most of these receptors are expressed in the primary sites where drugs of abuse act, such as nucleus accumbens (84), core/shell of nucleus accumbens (78), and ventral tegmental area (74). Very interestingly, these receptors are also expressed in vision system, such as retina (84), lacrimal apparatus(82), retinal pigment epithelium (84) and iris (82), which may help to explain the fact that drug abusers usually have the signs of unsteady gait bloodshot or watery eyes, dilated or constricted pupils (Silfies and DeMicco, 2013). It is well known that some drugs are illegally used for weight loss. For example, Clenbuterol, a β 2 agonist, has been off-label used as a weight-loss drug (Spiller et al., 2013). Actually, 83 of these 85 GPCR proteins, including β 2 adrenergic receptor, can be found in brown and white adipose tissues. Illegal drugs can affect fetus or infant (Prenatal Exposure to Drugs of Abuse, 2011), as these receptors are expressed in umbilical cord (83) and lactating mammary gland (79). Moreover, these receptors can also be found in islets of langerhans (81) and pancreas (78), evidence that abused drugs can disturb the functions of pancreas. This is consistent with the fact that drug use is associated with worse glycaemic control and higher risk of diabetic ketoacidosis (Lee et al., 2005, 2008, 2009, 2012; Lee and Campbell, 2008).

### Polypharmacology/polydrug addiction analysis for DA related GPCRs and their ligands

**Figure 3:**
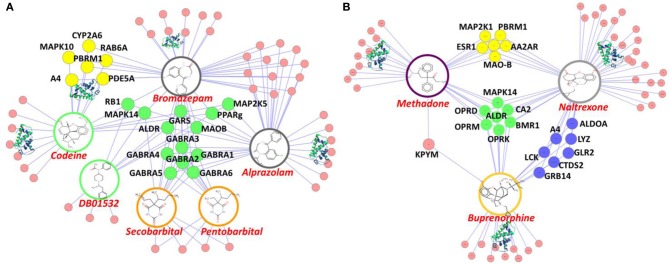
**Illustration of our cloud-based HTDocking server to predict potential targets (pink nodes) and cross-targets (yellow, green and blue nodes) of compounds and to explore possible mechanisms**. **(A)** The predicted targets of six known abused/approved drugs (*opioids*: codeine and DB01532; *benzodiazepines*: bromazepam and alprazolam; *barbiturates*: secobarbital and pentobarbital). **(B)** The predicted targets of 3 approved drugs for DA treatments (methadone, naltrexone, and buprenorphine). MAPK10, Mitogen-Activated Protein Kinase 10; CYP2A6, cytochrome P450, family 2, subfamily A, polypeptide 6; RAB6A, *RAB6A*, member RAS oncogene family; PBRM1, polybromo 1; PDE5A, phosphodiesterase 5A, cGMP-specific; A4, amyloid beta protein; RB1, retinoblastoma 1; MAPK14, mitogen-activated protein kinase 14; ALDR, Aldose reductase; PPARg, Peroxisome proliferator-activated receptor gamma; GABRA(1-6):Gamma-aminobutyric acid receptor subunit alpha-(1-6); MAOB, Monoamine Oxidase B; MAP2K1(5), Mitogen-Activated Protein Kinase 1(5); LCK, Tyrosine-protein kinase Lck; ESR1, Estrogen receptor; CTDS2, Carboxy-terminal domain RNA polymerase II polypeptide A small phosphatase 2; GRB14, Growth factor receptor-bound protein 14; GLR2, AMPA-selective glutamate receptor 2; ALDOA, Fructose-bisphosphate aldolase A; CA2, Carbonic anhydrase II; OPRD (OPRM,OPRK), Delta (Mu, Kappa)-type opioid receptor; AA2AR, Adenosine receptor A2a; MDR1, Multidrug resistance protein 1; GARS, Glycine-tRNA ligase.

To explore the potential interactions between DA/CNS-related drugs and proteins, we have applied our established HTDocking program for polypharmacology and polydrug addiction analysis in order to facilitate research of substance use disorders (SUDs), psychiatric or co-occurring disorders (CODs) through *in silico* molecular mechanism study. High prevalence of SUDs exists among those who have other mental illnesses and are vulnerable groups of polydrug or multiple DA use. Polydrug use often carries more risk than the use of a single drug, due to an increase in side effects and drug synergy. By exploring the molecular partners (or target proteins) of abused drugs, and further investigating the associated signaling pathways, we can elucidate the possible mechanisms of drug synergy, thereby contributing to the rational design of system pharmacotherapy and personalized medicines. As illustrated in Figure 3A, multiple targets (small colored nodes) of six abused drugs were predicted by our HTDocking program. These predicted top candidate targets (docking score >6.0) as well as the annotated known targets from DrugBank were compiled to build an interacting network or polypharmacology map. It is not a surprise that our plot shows both benzodiazepines and barbiturates can bind to GABA-A receptors (green nodes in Figure 3A), which may explain the synergized CNS depressants effects reported (Li and Xu, 2008). In fact, benzodiazepines are notorious for causing death when mixed with other CNS depressants such as opioids, alcohol, or barbiturates.

Among the predicted protein targets, our data show that PDE5A (yellow node, Figure 3A) is a target shared by codeine and bromazepam. It is well known that sildenafil (or Viagra), a famous PDE5A inhibitor, is used for sexual enhancement and can cause myocardial infarction (Cakmak et al., 2012). To our knowledge so far, there is no report about direct interactions between codeine and PDE5A except that some indirect evidence showed that codeine can induce sexual dysfunction (Ricardo Buenaventura et al., 2008). In addition, PPARγ (green node, Figure 3A) is the common target of three compounds: cocaine, bromazepam, and alprazolam. A drug targeting PPARγ for treatment of diabetes, e.g., rosiglitazone, is reported to have the side effect of myocardial infarction (Nissen and Wolski, 2007). Such information on polypharmacological effects will help us better evaluate the risk of polydrug use and of associated side effects, and could lead the way to discovery of new neurotherapy.

Another interesting *in silico* finding is the cross-target effects of three drugs (methadone, naltrexone, and buprenorphine). Our HTDocking study shows that besides binding to opioid receptors (OPRD and OPRM, green nodes), these three drugs are predicted to interact with carbonic anhydrase 2 (CA2, green node, Figure 3B). The finding is consistent with literature reports that drugs targeting CA2 can cause difficulty urinating (Roth et al., 1992), while difficulty urinating is one of the known adverse effects of methadone administration. As shown in Figure 3B, MOA-B (yellow node) is predicted as one of the common targets of methadone and naltrexone, while A4 (amyloid beta protein, blue node) is shared by naltrexone and buprenorphine. Considering that MOAB and A4 are also targets for management of Alzheimer's disease (AD), these predictions could lead to repurposing these drugs for AD treatment. The experimental validation of the computations-based predictions will be performed by our collaborators.

### Designing polypharmacology molecules targeting GPCRs for drug abuse treatment

**Figure 4:**
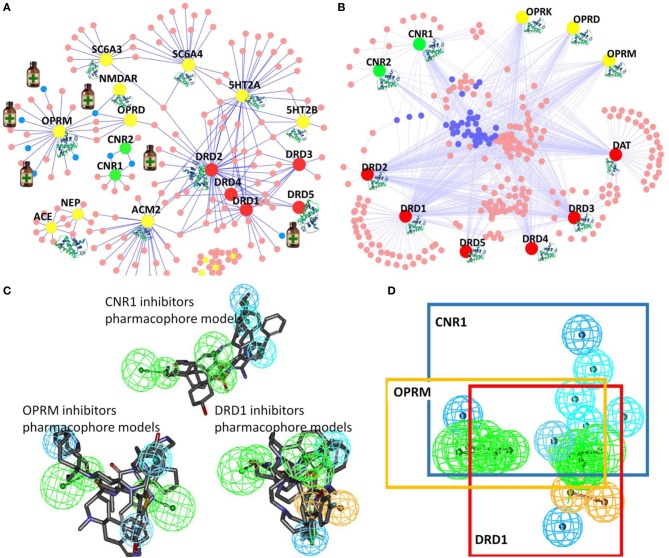
**Illustration of TargetHunter webserver (www.cbligand.org/TargetHunter) for polypharmacology DA research**. **(A)** TargetHunter mapping literature reported (abused) drugs-targets interactions. The large (yellow, green, and red) nodes represent targets. The small nodes (pink and blue) represent drugs, among which, seven, designated by medicine bottles (blue nodes), are approved/clinical trial medicine for DA treatments: (i) methadone, naltrexone, oxycodone, and buprenorphine targeting mu-opiate receptor (OPRM, large yellow nodes); (ii) Nabilone and Cannabidiol targeting cannabinoid receptor 1(CNR1 or CB1, large green nodes); and (iii) Adrogolide targeting dopamine receptor 1(DRD1, red). **(B)** TargetHunter prediction for possible cross-talk interactions of cannabinoid (CB) receptors (CNR1 and CNR2; green nodes), opiate receptors (OPRK, OPRD and OPRM; yellow) and dopamine receptors (DRD1, DRD2, DRD3, DRD4, and DRD5; red). Small blue and pink nodes in the center suggest potential cross-talk. **(C,D)** Pharmacophore models showing the shared common pharmacophoric features of the ligands of cannabinoid receptor 1 (CNR1), dopamine receptor D1 (DRD1) and mu-opioid receptor (OPRM).

In our *DA-KB*, we have collected data information on drugs and therapeutic agents for treatment of substance abuse disorders, as well as drugs for treatment of alcohol and tobacco use disorders, and their corresponding targets from data-mining literature reports and public/commercial databases. The current records consist of 163 drugs/therapeutic agents and 20 associated proteins. The computing polypharmacology network analyses of clinical trials or approved therapeutic medicines for DA treatment are illustrated in Figure 4A. The results show four out of the seven medicines, indicated by small medicine bottles target the mu-opioid receptor (OPRM, yellow), while two drugs interacting with the cannabinoid receptor 1 (CNR1, green), Nabilone and Cannabidiol, are in clinical trial for marijuana abuse (Phase II/III, NCT01347762) and opioid-related disorders (Phase II, NCT01605539), respectively. These are member of rhodopsin family GPCRs (Table 1).

We further applied our computational tools to explore the possibility of designing polypharmacological drug molecules targeting cannabinoid 1, mu-opioid and dopamine D1 for potential treatment of cocaine craving. As shown in Figure 4, our pilot study provides an interesting illustration for rational design of such polypharmacological ligands. By applying our TargetHunter program, we revealed that some cannabinoid ligands can interact with opioid receptors and dopamine receptors, which implied the possibility to design small molecules targeting cannabinoid receptor 1, dopamine receptors and mu-opioid receptor (Figure 4B). In addition, our established pharmacophore modeling (Chen et al., 2007) analysis showed that these small molecules shared common pharmacophoric features among these three receptors (Figures 4C,D). Such putative pharmacophore information will be highly useful for medicinal chemists to design and synthesize multiple-target ligands with synergetic potential for DA treatment. In addition, literature reported that dopamine ligands prevent the increases in ERK phosphorylation, which result from cocaine, amphetamine, methamphetamine, and THC administration. Thus, dopamine receptor could be a potential target for DA treatment (Lee and Messing, 2008). Moreover, our *in silico* prediction is also congruent with the report by Dong et al that the CB1-expressing neurons in the NAc are critical for emotional and motivational responses, and the membrane excitability of CB1-expressing fast-spiking interneurons within the NAc shell is increased after withdrawal from cocaine exposure, which may lead to increased release of GABA (Winters et al., 2012). Currently, we are carrying out medicinal chemistry synthesis to design/synthesis and biological test such multi-functional ligands with the functional pharmacophore groups that can simultaneously interact with the three GPCRs for DA treatment. The results will be reported in a separated journal.

## Conclusion

Taking together, we reported here a DA domain-specific chemogenomics knowledgebase repository system (DA-KB or CloudDA), including the database structure and data records as well as the implemented chemoinformatics tools/programs. With the illustrated DA polypharmacology networking analysis of GPCRs and drugs/therapeutic agents, we have demonstrated that DA-KB database can provide a powerful tool to transform the data to the knowledge related to DA research. The information will help to identify the characteristics and patterns of DA at the molecular and system pharmacology levels. Exploration of interactions between chemicals and DA proteins will allow better understanding of how genes/proteins and small molecules influence the various risks and protective factors for DA. Considering the current knowledge of GPCR distribution in the brain, these data may also help improve and expand our understanding of the brain circuitry that underlies DA. The impact will be broadened by our built cloud sourcing and cloud computing web service that can be accessed worldwide (www.CBLigand.org/CloudDA) and will certainly boost the data sharing and knowledge exchange among DA researchers and healthcare providers.

The polypharmacology effects of ligands of DA related GPCRs are very complex as showed in the preliminary analyses of cross-targets of opioids, benzodiazepines and barbiturates; target predictions of the 3 approved DA treatment drugs; and TargetHunter mapping literature reported (abused) drugs-targets interactions. The state-of-the-art computational technologies together with the chemogenomics database enable us to understand the DA in systems pharmacology level, for example, predicting the possible interactions with all the human proteins. Our TargetHunter and pharmacophoric study further suggested the possibility of finding and optimizing lead compounds that combine multiple desirable mechanisms of action on these GPCRs in a single new chemical entity for DA intervention.

Though our analyses focus on drug-abuse related GPCRs and their ligands, we believe such studies can be generalized to other DA related protein target families. As such, the DA-KB and associated tools will assist biologists to quickly derive the mechanisms of action for active chemicals used for DA treatment. They should help quicken target identification by prioritizing targets or target families according to the strength of the established associations with structural motifs found in small-molecule probes. They will also facilitate pharmaceutical scientists to do research for drug repurposing. Our developed algorithms and tools can predict new targets of approved drugs, thus allowing discovery of novel therapies for these drugs (particularly those that target the CNS) to fulfill unmet clinical needs. If overlap exists among the DA-related signaling pathways and those that are modulated by currently approved drugs, it is likely to repurpose such drugs for DA treatment. Given this insight, our methods will also address the needs of translational research that quickly translate basic “data” discoveries into new “knowledge” for clinical pharmacotherapy and personalized medicine (Collins, 2010).

### Conflict of interest statement

The authors declare that the research was conducted in the absence of any commercial or financial relationships that could be construed as a potential conflict of interest.
